# Wildfire worsens population exposure to PM2.5 pollution in the Continental United States

**DOI:** 10.21203/rs.3.rs-3345091/v1

**Published:** 2023-09-12

**Authors:** Danlu Zhang, Wenhao Wang, Yuzhi Xi, Jianzhao Bi, Yun Hang, Qingyang Zhu, Qiang Pu, Howard Chang, Yang Liu

**Affiliations:** Emory University; Emory University; Emory University; Emory University; Emory University; Emory University; Emory University; Emory University

**Keywords:** Smoke PM2.5, wildfire, air pollution, remote sensing, machine learning

## Abstract

As wildfires become more frequent and intense, fire smoke has significantly worsened ambient air quality, posing greater health risks. To better understand the impact of wildfire smoke on air quality, we developed a modeling system to estimate daily PM_2.5_ concentrations attributed to both fire smoke and non-smoke sources across the Continental U.S. We found that wildfire smoke has the most significant impact on air quality in the West Coast, followed by the Southeastern U.S. Between 2007 and 2018, fire smoke affected daily PM_2.5_ concentrations at 40% of all regulatory air monitors in EPA’s Air Quality System (AQS) for more than one month each year. People residing outside the vicinity of an EPA AQS monitor were subject to 36% more smoke impact days compared to those residing nearby. Lowering the national ambient air quality standard (NAAQS) for annual mean PM_2.5_ concentrations to between 9 and 10 μg/m^3^ would result in approximately 29% to 40% of the AQS monitors falling in nonattainment areas without taking into account the contribution from fire smoke. When fire smoke impact is considered, this percentage would rise to 35% to 49%, demonstrating the significant negative impact of wildfires on air quality.

## Introduction

With a changing climate, large-scale wildfire events have increased in frequency and intensity, and fire seasons have been prolonged in the Contiguous U.S. (CONUS) in recent decades (1, 2). Wildfire smoke contains large quantities of fine particulate matter (PM_2.5_, airborne particles with diameters smaller than 2.5 μm), and can adversely affect regional air quality in downwind communities that are tens to hundreds of kilometers away. For instance, Jaffe et al. (2008) reported that PM_2.5_ levels have increased in summer due to wildfires in the western U.S. (3) and Geng et. al observed an significant enhancement in PM_2.5_ concentrations in intensive wildfire years in Colorado (4). This impact has become so expansive that a previous analysis of PM_2.5_ measurements from U.S. EPA’s ground monitoring network between 1988 and 2016 attributed the increasing trend of 98th quantile of 24-hour PM_2.5_ concentration in the Northwestern U.S., in contrast to the decreasing trend in the rest of the Continental U.S., to the influence of wildfires (5). In January 2023, the U.S.EPA proposed to revise the National Ambient Air Quality Standards (NAAQS) of PM_2.5_ by lowering the primary annual PM_2.5_ standard to the range of 9.0 to 10.0 μg/m^3^ (6). As both fire season length and wildfire frequency are projected to increase worldwide and particularly in North America under both 1.5°C and 2.0°C global warming targets (7), attainment under the new annual PM_2.5_ standard will be more challenging in fire prone regions.

Studies worldwide have reported significant associations of both acute and chronic exposure to ambient PM_2.5_ with various adverse health outcomes including respiratory and cardiovascular diseases, nervous system diseases, and premature mortality (8–10). Smoke PM_2.5_ contains 5–20% elemental carbon (EC) and at least 50% organic carbon (OC) including many polar organic compounds (11). The greater oxidative potential of smoke PM_2.5_ suggests a different if not greater toxicity than ambient PM_2.5_. In addition, with the expanding wildland-urban interface and an aging U.S. population, the overall burden of wildfire-related diseases is expected to increase (12). A few previous studies have linked acute exposure to wildfire smoke PM_2.5_ with a series of adverse health outcomes (13). For example, Alman et al. (2016) positively associated short-term exposure of PM_2.5_ from wildfire with respiratory illnesses (14). Stowell et al. (2019) reported significant association between smoke PM_2.5_ exposure and a greater risk of emergency department visits due to asthma attacks after controlling for PM_2.5_ exposure from non-smoke sources in Colorado (15).

While chronic exposure to ambient PM_2.5_ has been shown to present a much greater risk to human health than acute exposure (9), few studies have assessed the health effects of chronic wildfire smoke PM_2.5_ exposure primarily due to the challenge of estimating long-term wildfire smoke PM_2.5_ exposure at high spatial and temporal resolutions. Since most wildfires started in remote areas, regulatory monitoring networks such as US EPA’s Air Quality System (AQS) are often insufficient to characterize the spatial patterns of smoke PM_2.5_. In addition, ground observations alone cannot separate fire smoke PM_2.5_ from other sources. Chemical transport models (CTMs) such as the Community Multiscale Air Quality (CMAQ) Model can simulate fire-specific PM_2.5_ with full coverage in space and time, greatly expanding the study population of air pollution epidemiological studies to cover both urban and rural populations (4). However, uncalibrated CTM smoke simulations frequently suffer from substantial prediction errors caused by imperfect characterization of complex fire chemistry, inaccurate emission inventory and rapidly changing local meteorology surrounding fires (16). Most recently, machine learning or statistical models that integrated ground observations, satellite remote sensing data, land cover land use information, as well as CTM simulations have shown great promise to generate long-term, accurate and high-resolution ambient PM_2.5_ concentrations worldwide with full spatial and temporal coverage. To date, a handful of non-CTM-based fusion models to estimate smoke PM_2.5_ levels have been reported. For example, O’Dell et al. (2019) estimated the contribution of wildland-fire smoke to seasonal mean PM_2.5_ levels in the CONUS at a spatial resolution of ~ 15 km (17). Childs et al. (2020) estimated daily smoke PM_2.5_ concentrations at 10 km spatial resolution using satellite-based fire smoke contours to define fire days. The coarse spatial resolutions of these studies cannot capture the detailed spatial gradients of smoke PM_2.5_ levels. The lack of ground observations near the fires to be included in model training is also attributable to the underestimation of peak smoke PM_2.5_ concentrations in these studies.

Here, we designed a multi-stage, CTM-based modeling framework to estimate full-coverage, daily smoke PM_2.5_ concentrations in the CONUS at 1 km spatial resolution. This framework integrated CMAQ PM_2.5_ simulations, multiple satellite remote sensing products, meteorology reanalysis, land cover land use information, and ground observations from both regulatory and low-cost sensor networks. Taking advantage of the high spatial and temporal resolution of our model predictions, we investigated the long-term impact of wildfires on national air quality as well as the representativeness of AQS monitoring network in estimating population exposure to fire smoke. In addition, we investigated the impact of lowering the PM_2.5_ standard on the attainment areas and the number of individuals affected by it, both with and without the influence of smoke emissions from fires.

## Materials and Methods

### Ground PM_2.5_ Measurements and Calibrations

We obtained Environmental Protection Agency (EPA) federal reference and acceptable ground PM_2.5_ measurements which were publicly available at the AQS (18). We calculated daily PM_2.5_ concentrations by averaging the hourly measurements at stations and days with at least 16 out of 24 possible measurements. The rapidly developing low-cost sensor networks are a significant supplement of traditional monitoring due to their high spatial density and temporal frequency (19, 20). We included measurements from the PurpleAir low-cost PM_2.5_ sensors to extend the spatiotemporal coverage of ground monitoring and increase the probability of capturing the PM_2.5_ pollutions from wildfire smoke (21, 22). The PurpleAir is a citizen-based real-time PM_2.5_ monitoring network with nearly 10,000 sensors currently online globally (23). By utilizing measurement calibration methods (24, 25), previous studies suggested that the low-cost sensor can be a significant supplement to the reference ground monitors in PM_2.5_ exposure assessments (26, 27). Vu et al, incorporated the PurpleAir network with AQS monitors in estimating regional PM_2.5_ levels during a fire event in California (28). Since the PurpleAir PM_2.5_ measurements have bias with the reference-grade measurements, we performed a series of quality control and calibration (29). We first removed all station-days with less than 16 hourly measurements and those with 30 percent relative difference among two channels. We also removed extreme measurements with daily value more than 1,000 μg/m^3^, temperature less than − 20 F° or higher 140 F°, and humidity less than 0% or higher than 100%. We conduct Geographically Weighted Regression (GWR) to calibrate PurpleAir measurements which is similar to many previous studies (26). In order to perform calibration widely among the entire study domain, we matched PurpleAir monitors and AQS stations within 5 km buffers and a total of 230 AQS stations were paired but around half of paired stations are located in west U.S. Since relative humidity and temperature have great impact on PurpleAir accuracy (26), we divided CONUS into 4 parts to balance the climate regions and paired stations locations, as shown in Figure S1. We developed four regional GWR models, with relative humidity and temperature to calibrate the PurpleAir measurements. The 20-km buffer was created for each region and calibrated PurpleAir observations located in buffers were calculated as the mean of two GWR models’ outputs in order to make a smooth transition between regions. Calibrated daily PurpleAir observations over annual standard of 12 μg/m^3^ were added in our final model.

### Data Integration

A large array of predictor variables was used to develop the PM_2.5_ models, including satellite-retrieved aerosol, cloud, and smoke plumes information, gridded meteorology, population, land cover and topographic data (detailed descriptions provided in the Supplementary Materials).

All data sets at various spatial resolutions were integrated at the 1-km grid of the Multi-Angle Implementation of Atmospheric Correction (MAIAC) Aerosol Optical Depth (AOD). Due to the missing data issue in MAIAC AOD, we applied a two-step gap-filling approach to obtain a full coverage MAIAC AOD (detailed descriptions provided in the Supplementary Materials). Daily average PM_2.5_ measurements from the AQS monitors and PurpleAir sensors were assigned to their collocated grid cells and averaged PM_2.5_ measurements were calculated at grid cells with multiple monitors. Note that the PurpleAir data were calibrated based on a previously reported method before merging with AQS measurements (26). We interpolated the coarse resolution variables into 1-km resolution using inverse distance weighting (30). They include CMAQ, Copernicus Atmosphere Monitoring Service (CAMS) AOD and meteorological factors. We obtained the land cover data at 30-meter resolution is obtained from the National Land Cover Database. We collected road network and elevation data from the Global Roads Inventory Project and the Global Digital Elevation Model Version 3, respectively. For each grid cell, we calculated the percentages of land cover types, average elevation, and total road length. We matched our grid with the 1-km resolution population density data, which is from the Landscan Program at Oak Ridge National Laboratory (ORNL) (31). We calculated daily total smoke plumes duration, daily weighted average plume density for each grid cell using the fire smoke polygons produced by the National Oceanic and Atmospheric Administration (NOAA) Hazard Mapping System (32, 33). Terra and Aqua Moderate Resolution Imaging Spectroradiometer (MODIS) cloud fractions at 5 km resolution were assigned to the overlapped grid cells, and then averaged if available. Based on climate types, CONUS is divided into nine climate regions (34) and indicators of climate region were assigned to the overlapped grid cells.

### Smoke PM_2.5_ Model Development

Random Forest (RF) is an ensemble algorithm based on multiple decision trees and the outputs from all decision trees are averaged to be the prediction of the dependent variable (35, 36). Each decision tree is built on a bootstrap training data and a subset of independent variables are randomly selected in each tree node (35). The bootstrap strategy allows RF to be a robust model against overfitting (36). RF also provides an estimated importance rank which informs the weights of predictors and allows an easier interpretation, comparing with neural network models (35, 37). The R^2^ and Root Mean Squared Error (RMSE) were calculated from overall, spatial and temporal 20-fold cross-validation (CV) and we used them to assess the model performance and furthermore adjust model parameters.

**Figure 1:**
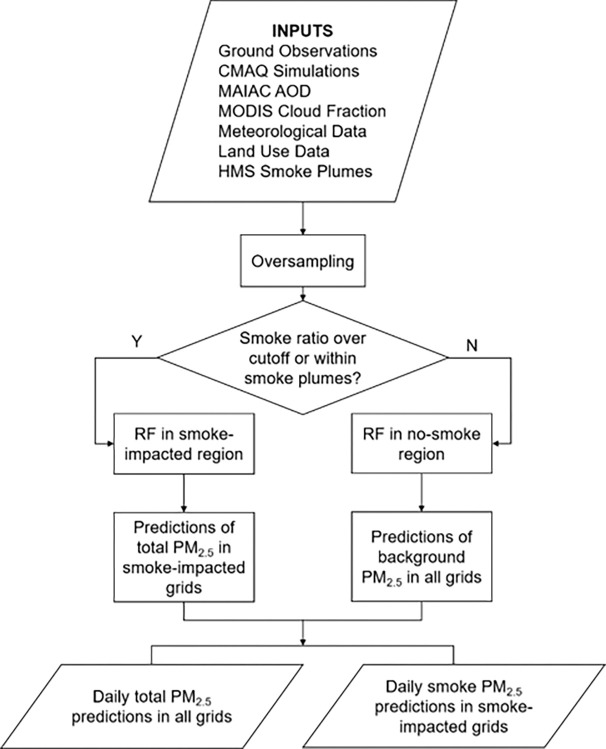
Flow diagram of the PM_2.5_ modeling framework.

Two random forest algorithms were trained independently in order to separate smoke PM_2.5_ from the background PM_2.5_ (Fig. 1). First, the modeling grid cells and days were divided into smoke-impacted regions and no-smoke regions according to daily HMS smoke plume polygons and the CMAQ smoke ratio (i.e., simulated smoke PM_2.5_ over total PM_2.5_ concentration). A smoke grid cell was defined as either being inside an HMS smoke plume polygon or having a CMAQ smoke ratio greater than 0.03 on a given day. Next, in the smoke-impacted region, a random forest algorithm was trained to estimate daily total PM_2.5_ concentrations, which was assumed to be the sum of smoke contribution and background (i.e., contribution from all the other sources). In the no-smoke region, smoke contribution was assumed to be negligible and a separate random forest algorithm was trained to estimate daily background PM_2.5_ concentrations in the no-smoke region. Then, this no-smoke algorithm was also used to predict daily background PM_2.5_ concentrations in the smoke-impacted region. Finally, the daily smoke PM_2.5_ concentration in each grid cell of the smoke-impacted region was then calculated as the difference between predicted total PM_2.5_ concentration and the predicted background PM_2.5_ concentration. Since only a small proportion of extreme-high ground PM_2.5_ concentrations were captured by AQS data, we applied a Synthetic Minority Over-sampling Technique (SMOTE) to oversample the underrepresented measurements with high levels to improve the model performance at high PM_2.5_ concentrations (28, 38). SMOTE generated synthetic samples along with their predictions from the five nearest neighbors in the training dataset (38). PM_2.5_ concentrations over 35 (U.S. NAAQS for 24-hour PM_2.5_) and below 100 μg/m^3^ were oversampled once while the PM_2.5_ measurements over 100 μg/m^3^ were oversampled twice through SMOTE. The oversampled data accounted for 0.85% of the total input data, and the SMOTE process did not skew the distribution of PM_2.5_ observations. Our final training dataset for smoke-impacted and no-smoke models had 1,657,449 and 2,003,085 station-day observations, respectively.

The formulas of models in smoke-impacted and no-smoke regions are:

$$\mathrm{modelinno}\;-\mathrm{smokeregion}\;:\;PM_{\left(s,t\right)}=PM_{B\left(s,t\right)}=f\left(X_{\left(s,t\right)},Z_{\left(s,t\right)}\right)$$

$$\mathrm{modelinsmoke}\;-\;\mathrm{impactedregion}\;:\;PM_{\left(s,t\right)}=PM_{F(s,t)}+PM_{B(s,t)}=f(X_{\left(s,t\right)}^{'},Z_{\left(s,t\right)}^{'})$$

where $PM_{\left(s,t\right)},PM_{F(s,t)}\;$ and $PM_{B(s,t)}\;$ denote the ground measured PM_2.5_ concentration, fire component PM_2.5_ and non-fire background PM_2.5_ at location s on day t, respectively. For the model in no-smoke region, $X_{\left(s,t\right)}$ is the CMAQ simulated background PM_2.5_ at location s on day t, and $Z_{\left(s,t\right)}\;$ is a vector of additional predictors, including gap-filled MAIAC AOD, meteorological factors, cloud fractions, land cover and climate region types, as listed in Table S1. For the model in smoke-impacted region, $X_{\left(s,t\right)}^{'}\;$ is the CMAQ simulated total PM_2.5_ at location s on day t, while $Z_{\left(s,t\right)}^{'}\;$ includes the HMS data and all predictors in $Z_{\left(s,t\right)}.$

## Results and Discussion

### Model Performance

The R^2^ of overall, spatial and temporal CV of smoke-impacted model is 0.75 (RMSE = 4.59 μg/m^3^), 0.59 (RMSE = 5.88 μg/m^3^) and 0.67 (RMSE = 5.18 μg/m^3^), respectively, indicating a good model performance in fire grids. For no-smoke model, the R^2^ of random, spatial and temporal cross validation is 0.68, 0.47 and 0.63, with RMSE of 3.35 μg/m^3^, 4.30 μg/m^3^ and 3.59 μg/m^3^, respectively, which indicates the satisfactory performance from the random forest model for background PM_2.5_. As shown in Figure S2, random forest models slightly overestimated at low PM_2.5_ concentrations and underestimate at high PM_2.5_ values, especially when daily PM_2.5_ concentration exceeds 100 μg/m^3^. After aggregating the daily PM_2.5_ predictions to monthly level, the R^2^ of smoke-impacted and no-smoke models in the overall 20-fold CV increased to 0.84 and 0.78, respectively, indicating the bias of estimation is random. Scatter plots for aggregated monthly CV are shown in Figure S3. Same process was used for spatial and temporal CV and as a result, the R^2^ of both smoke-impacted and no-smoke models were improved, as shown in Table S2. After aggregating the overall CV to annual level, the R^2^ between all predictions and AQS measurements is 0.9, implying a high accuracy of model predictions. As for variable importance, CMAQ is the most important predictors in both smoke-impacted and no-smoke and AOD and wind are the common parameters ranked in top five in two models (Figure S4).

### Spatiotemporal Patterns of Smoke PM_2.5_ across the CONUS

**Figure 2:**
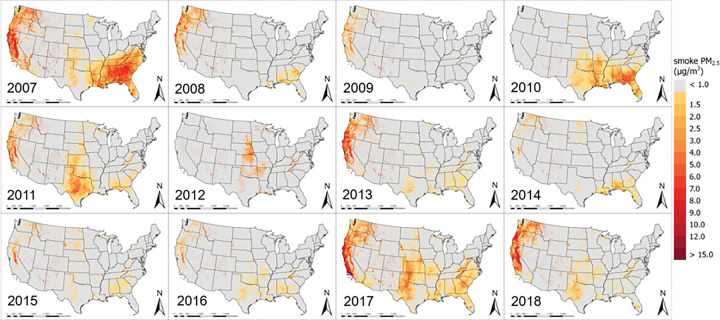
Annual mean smoke PM_2.5_ concentration (μg/m^3^) from 2007 to 2018 in the CONUS.

Figure 2 presents spatial distributions of annual mean smoke PM_2.5_ in the CONUS from 2007 to 2018. While the Western U.S. has seen a significant and more persistent impact of fire smoke on PM_2.5_ levels, other regions including the mid-West and the Southeast have also suffered high smoke PM_2.5_ in certain years. For example, annual average smoke PM_2.5_ concentrations over 8 μg/m^3^ occurred in California, Oregon, and Washington in 2007–2009, 2011, 2013, 2017 and 2018, and over 50% of the areas in these states were impacted by fire smoke during these years. Along California coasts and in the Central Valley, annual average smoke PM_2.5_ concentrations exceeded 12 μg/m^3^ in 2007, 2017 and 2018. We observed the highest annual average wildfire smoke PM_2.5_ level north of Ventura County in Southern California at 25 μg/m^3^ in 2017. Other Western states such as Idaho, Montana, Utah, Colorado, Arizona, and New Mexico have been affected to a lesser degree, with annual mean smoke PM_2.5_ levels ranging between 0 and 5 μg/m^3^. The second most affected region by fire smoke is the Southeast. For example, annual smoke PM_2.5_ levels up to 9 μg/m^3^ were common in Alabama, Georgia, and the Carolinas. Fire smoke also contributed significantly to elevated PM_2.5_ levels in Georgia and Florida in 2010 and 2017. In addition, air quality in the Midwestern states was periodically affected by fire smoke. For example, approximately half of Texas, Oklahoma and Kansas showed detectable fire smoke impact in 2010, 2011, and 2017, with high smoke PM_2.5_ levels observed over large cities such as Dallas, Austin and San Antonio. PM_2.5_ levels in the states around the Great Lakes and in the Northeastern U.S. have rarely been affected by fire smoke during our study period.

Conducting large-scale epidemiological studies to investigate the impact of fire smoke on human health has been challenging largely due to the difficulty in estimating spatially resolved exposure to fire smoke PM_2.5_. Recently, a few modeling studies of smoke PM_2.5_ concentrations in the CONUS have been conducted with spatial resolutions ranging from 10–15 km (17, 39). Using machine-learning models such as those presented in this study allows the integration of CTM fire simulations, high-resolution satellite remote sensing of fire smoke, and the broader spatial representation of the PurpleAir sensor network to achieve high spatial resolution (1 km), high temporal resolution (daily), and full coverage of the CONUS for a 12-yr period. The temporal trend and spatial characteristics of our model-predicted smoke PM_2.5_ concentrations align with major fire events across the country. For example, data from the National Interagency Fire Center (40) showed that fire activities in Southern California, eastern Texas, and southern North Carolina and Tennessee in 2007 were 125% and 121% of previous 10-year average, respectively. The acres burned in the Rocky Mountains were 367% and 351% of previous 10-year average in 2012 and 2017, respectively, and our model successfully capture these features. Compared with uncalibrated CMAQ simulations of smoke PM_2.5_ (Figure S5 Panel A), our predictions better represent the spatial and temporal distribution of fire smoke. For instance, our model captured the high smoke PM_2.5_ values in the West and Southeast during the extreme fire years, such as 2007 and 2018 (Fig. 1), and low smoke PM_2.5_ values in 2015, which have same temporal trend as reported by National Interagency Fire Center (40). In addition, our model was able to capture finer spatial features more accurately due to its high spatial resolution at 1 km. Compared with previous smoke PM_2.5_ estimations with coarse resolution, our predictions provided a clearer boundary of the smoke impacted areas and captured detailed variability of population exposure levels. As illustrated in Figure S6, population within an area of 100 km^2^ in Sacramento, California were able to be assigned to 100 unique smoke PM_2.5_ values based on their locations rather than one average value, which offers the feasibility for high-resolution health impact studies.

To our best knowledge, our study is the first large-scale attempt to use calibrated PM_2.5_ concentration measurements from low-cost sensors such as PurpleAir monitors in conjunction with AQS monitors to better characterize the spatial variability of smoke PM_2.5_. Previous research has shown that low-cost sensor measurements can increase the likelihood of detecting wildfire smoke (21, 22), and integrating low-cost sensor data with regulatory measurements has allowed for better training of satellite-based machine learning models for identifying air pollution hotspots (26, 41). In our study, PurpleAir sensors reported extreme PM_2.5_ concentrations over 200 μg/m^3^ during the Camp fire in California, while the highest AQS measurement was approximately 100 μg/m^3^ as there were no AQS monitors located near the smoke plumes. Including the high PM_2.5_ measurements from PurpleAir in our training dataset reduced the model underestimation on high PM_2.5_ values. For instance, the smoke PM_2.5_ prediction from models without PurpleAir (Figure S5, Panel B) was biased low in California where high smoke PM_2.5_ values always occurred and the difference of annual smoke PM_2.5_ predictions between models with and without PurpleAir measurements reached up to 16 μg/m^3^ in 2018. Unlike earlier studies which attributed the deviation from background levels of PM_2.5_ to smoke using ground total PM_2.5_ measurements, satellite-based smoke plume identification, and air trajectories (17, 39), we employed two different CMAQ simulations, with and without fire emissions, along with satellite-based HMS smoke contours to more accurately label smoke impacted areas and days. Our approach facilitates independent modeling of both background PM_2.5_ and total PM_2.5_ accounting for smoke impact nationwide.

### Effect of Fire Smoke on National PM_2.5_ Concentration Levels

**Figure 3:**
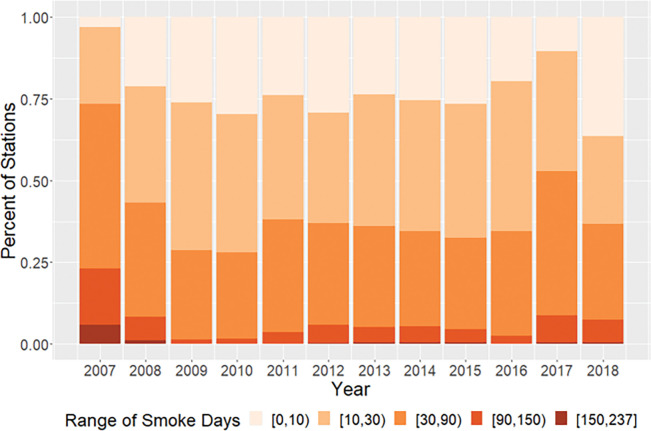
Fractions of EPA PM_2.5_ monitoring locations significantly affected by fire smoke from 2007 to 2018.

Using our daily model predictions, we assessed the impact of fire smoke on the regulatory air quality monitoring network. We defined a smoke impact day as when fire smoke contributed more than 25% of model-estimated daily total PM_2.5_ mass concentration at the location of an air quality monitoring station included in the EPA AQS. Daily PM_2.5_ concentration at ~ 40% of the 1836 AQS monitoring sites have been significantly affected by smoke for more than a month each year during our study period (Fig. 3). In 2009 and 2010 when our model predicted the lowest smoke impact on national PM_2.5_ levels, over 25% of the national ambient PM_2.5_ monitoring network was under significant smoke impact for more than a month. In intensive fire years such as 2017, 50% of all monitoring locations were affected for at least a month, indicating a widespread impact at the national scale. During the worst fire year of 2007, 25% of all monitoring locations were affected for more than 90 days. Smoke impact on air quality was highest in summer and fall in most years. However, in low fire years such as 2009 and 2010, fire smoke had the greatest impact in spring and fall.

### AQS’s Representativeness of Population Exposure to Fire Smoke

Using our model predictions and annual population estimates at 1 km resolution, we estimated the U.S. population affected by fire smoke. As shown in Table 1, nearly the entire population in the CONUS, ranging from 95% in 2018 to 100% in 2007, has been exposed to fire smoke. On average, a slightly higher percentage of people living outside the vicinity of an EPA AQS monitoring station (defined by a 5 km radius) has been exposed to fire smoke. The average duration of population exposure to fire smoke showed a more substantial difference. On average, people living outside the vicinity of an AQS monitoring station experienced 25.2 smoke impact days, 36.5% (ranging from − 8% in 2018 to 70% in 2012) greater than people living near an AQS station. While the mean model estimated total PM_2.5_ concentration in regions near an AQS station (10.79 μg/m^3^) is significantly higher than that in regions without AQS coverage (8.87 μg/m^3^), estimated smoke PM_2.5_ concentration shows the opposite (0.50 μg/m^3^ vs. 0.65 μg/m^3^). Since the majority of AQS stations are located in urban areas, these findings suggest that using EPA observations alone may substantially underestimate both the duration and the concentration of the fire smoke exposure of the rural and suburban population.

### Impact of Fire Smoke on Attainment Status with the Proposed New PM_2.5_ Standard

In January 2023, the U.S. EPA proposed to lower the NAAQS for annual mean PM_2.5_ concentrations, calculated as the average of past three years, to a value between 9 μg/m^3^ and 10 μg/m^3^. We estimated the total population as well as the number of AQS monitoring sites which would reside in nonattainment areas under the new standard (Table S3 and S4). Without considering in the impact of fire smoke, an average of 116.83 million people (from 68.73 million in 2016 to 148.74 million in 2013) and 30% of all AQS monitoring sites (from 15% in 2017 to 40% in 2011) in the CONUS would be in areas with annual mean PM_2.5_ concentrations equal to or above 10 μg/m^3^. When we considered the fire smoke contribution to PM_2.5_ levels, an additional 21.4 million people and 6% of AQS monitors would reside in nonattainment areas. Under the stricter standard of 9 μg/m^3^, the average affected population would increase to 167.23 million without considering the effect of fire smoke, and 197.68 million (ranging from 153.73 million in 2016 to 225.27 million in 2013) with the contribution of fire smoke. Regarding air quality monitoring, an average of 41% of all AQS monitoring sites would fall into nonattainment areas. When the contribution of fire smoke was considered, this percentage rose to 50% (ranging from 37% in 2016 to 58% in 2011 and 2012).

As the increasing regulation of emissions of PM_2.5_ and its precursors from anthropogenic sources have effectively improved air quality in most parts of the US, fire emissions are becoming a major contributor of PM_2.5_. The proximity of large populations to wildland fires poses a nontrivial threat to public health and compliance with ambient air quality standards. According to EPA (42), approximately 20.9 million Americans (2010 population) reside in PM_2.5_ nonattainment areas based on the current NAAQS as of 2023. Our model estimated that 95.9 to 146.3 million more people would live in nonattainment areas if the annual mean PM_2.5_ NAAQS were lowered to between 9 and 10 μg/m^3^. Our calculations also suggested that taking the impact of fire smoke into account would result in an additional 21.4 to 30.5 million people falling into nonattainment areas. As most wildland fires start in rural areas, fire smoke PM_2.5_ would disproportionally affect the suburban and rural populations. The comprehensive spatial coverage of our model estimates would enable future research on the differential health effects of air pollution exposure associated with the altered PM_2.5_ composition in these communities.

## Figures and Tables

**Table 1:** Fire smoke impact on the U.S. population.

Year	Total Population (Population without AQS coverage) (million)	Smoke Impacted Total Population (Smoke Impacted Population without AQS coverage) (million)	Smoke Impact Days among Population with AQS coverage (among Population without AQS coverage)	Total PM_2.5_ (Smoke Pm_2.5_) with AQS coverage	Total PM_2.5_ (Smoke PM_2.5_) without AQS coverage
2007	300.1 (73.6)	299.7 (73.6)	38.2 (54.6)	11.90 (0.96)	9.87 (1.11)
2008	302.6 (74.1)	298.3 (72.6)	21.2 (22.4)	10.42 (0.32)	8.26 (0.38)
2009	305.5 (70.9)	300.3 (69.9)	13.5 (19.4)	11.20 (0.25)	8.45 (0.22)
2010	307.0 (72.0)	285.7 (71.6)	12.8 (22.6)	10.83 (0.57)	9.73 (0.77)
2011	310.0 (72.9)	307.9 (72.7)	16.4 (25.7)	11.43 (0.51)	9.14 (0.73)
2012	299.9 (72.0)	289.0 (71.6)	11.9 (20.3)	10.35 (0.53)	9.28 (0.83)
2013	313.1 (74.0)	308.0 (72.9)	16.7 (19.1)	11.57 (0.61)	9.34 (0.66)
2014	317.3 (74.6)	310.8 (74.3)	16.9 (22.5)	9.40 (0.31)	8.74 (0.40)
2015	319.8 (74.9)	313.2 (74.6)	14.4 (19.2)	9.37 (0.48)	7.91 (0.64)
2016	321.5 (74.9)	319.7 (74.9)	17.6 (25.0)	9.30 (0.31)	7.98 (0.48)
2017	324.1 (74.6)	321.6 (74.5)	26.2 (33.8)	11.22 (0.97)	8.78 (0.92)
2018	325.6 (74.8)	308.9 (73.1)	20.2 (18.5)	10.51 (0.61)	8.95 (0.65)
Average	312.2 (73.6)	305.3 (73.0)	18.8 (25.2)	10.79 (0.50)	8.87 (0.65)
